# Robust Human Tracking Using a 3D LiDAR and Point Cloud Projection for Human-Following Robots

**DOI:** 10.3390/s25061754

**Published:** 2025-03-12

**Authors:** Sora Kitamoto, Yutaka Hiroi, Kenzaburo Miyawaki, Akinori Ito

**Affiliations:** 1 Graduate School of Robotics and Design, Osaka Institute of Technology, Osaka 530-8568, Japan; m1m23r14@st.oit.ac.jp; 2 Faculty of Robotics and Design, Osaka Institute of Technology, Osaka 530-8568, Japan; yutaka.hiroi@oit.ac.jp; 3 Faculty of Information Sciences and Technology, Osaka Institute of Technology, Hirakata 573-0196, Japan; kenzaburo.miyawaki@oit.ac.jp; 4 Graduate School of Engineering, Tohoku University, Sendai 980-8579, Japan

**Keywords:** 3D LiDAR, human tracking, human-following robot

## Abstract

Human tracking is a fundamental technology for mobile robots that work with humans. Various devices are used to observe humans, such as cameras, RGB-D sensors, millimeter-wave radars, and laser range finders (LRF). Typical LRF measurements observe only the surroundings on a particular horizontal plane. Human recognition using an LRF has a low computational load and is suitable for mobile robots. However, it is vulnerable to variations in human height, potentially leading to detection failures for individuals taller or shorter than the standard height. This work aims to develop a method that is robust to height differences among humans using a 3D LiDAR. We observed the environment using a 3D LiDAR and projected the point cloud onto a single horizontal plane to apply a human-tracking method for 2D LRFs. We investigated the optimal height range of the point clouds for projection and found that using 30% of the point clouds from the top of the measured person provided the most stable tracking. The results of the path-following experiments revealed that the proposed method reduced the proportion of outlier points compared to projecting all the points (from 3.63% to 1.75%). As a result, the proposed method was effective in achieving robust human following.

## 1. Introduction

Human tracking is a fundamental technology for mobile robots that work with humans. Various devices are used for human tracking, such as cameras with computer vision technology [1,2,3], RGB-D sensors [4,5,6], millimeter-wave radar [7,8,9], and laser range finders [10,11]. Observations from multiple sensors are often combined [12,13,14].

Laser range finder (LRF)-based human tracking has several advantages over methods that use other sensors. Measurements using LRFs are more robust to lighting conditions, more accurate than camera-based methods, and require less computation than camera-based measurements. A drawback of LRF-based measurement is that an ordinary LRF observes only a specific horizontal plane, which can cause misdetection of the human body because the measurement plane may miss the target position (such as the torso) when the height of the human varies.

A 3D LiDAR (Light Detection and Ranging) [15] is a laser-based sensor that measures distances to objects across multiple planes. Several studies have used a 3D LiDAR as a human detection and tracking sensor because it can capture the surface of surrounding objects as point clouds [16,17,18]. However, using the observed point cloud directly for human tracking requires high computational costs.

Here, we develop an energy-efficient human-tracking method for a small mobile robot that follows a person. We have already developed a simple, robust, and computationally low-cost human-tracking method based on 2D LRFs [19,20]. Therefore, this paper proposes a technique that projects the point cloud onto a 2D plane to apply the 2D-LRF-based human-tracking method and improve robustness to human height variations.

This paper is organized as follows. We investigate related works in Section 2 and explain the proposed method in Section 3. In Section 4, we describe two experiments: objective and subjective evaluations. In Section 5, we conclude this paper.

## 2. Related Works

### 2.1. Human Tracking Using 2D LRFs

A number of human-tracking methods using LRFs have been proposed so far. The method by Fod et al. was one of the earliest works [21]. Their work first detects objects in the observation plane as blobs and then merges the blobs of the same person as a post-processing step. Other methods aim to either detect and track humans with lightweight processing for low-computational-resource devices [22,23,24] or use machine learning/deep neural networks for accurate detection and tracking [25,26,27].

When tracking humans, an LRF can only observe one horizontal plane. Therefore, it is important to determine the appropriate measurement height of the LRF. Small robots often observe human legs [22,23]; however, the detection of human legs is often unstable, especially in environments where there are multiple people.

**Figure 1:**
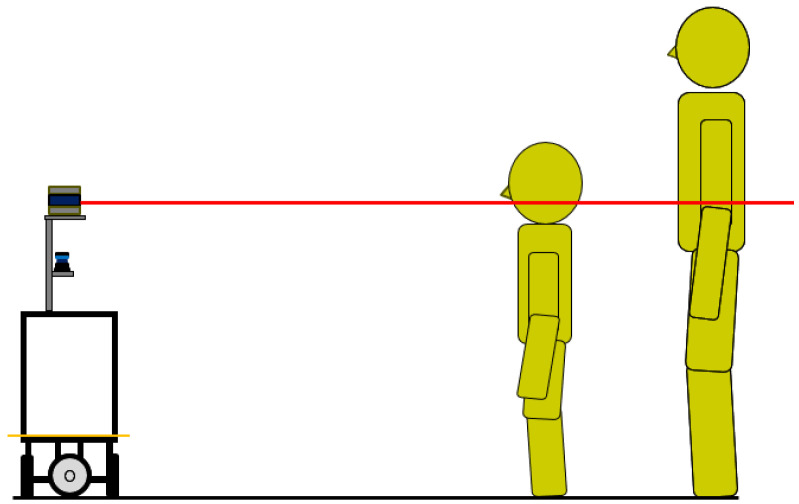
Observation of humans using a 2D LRF. Since the LRF observes the environment at a specific height, it captures different parts of the body depending on a person’s height.

Tracking the torso is another common method in LRF-based human tracking [24,25]. There are two problems with torso detection: first, it is affected by arm movements, and second, its performance depends on the person’s height. In particular, detecting shorter people, such as children, is challenging, as shown in Figure 1.

Our previous work belongs to the latter, where the human torso is detected and tracked using an LRF [24]. This method is stable, robust, lightweight, and suitable for mobile robots. A similar method can detect and track multiple people moving quickly around the robot [19,20].

More recent studies have also applied LRFs for human tracking. Hasan et al. proposed a person identification technique named “PerFication” that utilizes LRF data to evaluate gait. The system uses ankle-level LRF data for individual tracking and identification, especially in environments where video monitoring is ineffective [28]. Aguirre et al. developed a method that combines observations from an LRF and a vision-based deep learning technique [27,29]. Kutyrev et al. applied an LRF to trajectory control for autonomous vehicles used in a horticultural plant field [30].

When observing humans using an LRF, the height of the LRF should be adjusted to match the height of the humans being tracked. For example, we previously demonstrated a robot that tracked humans at events such as our institute’s open house [31], where many children visited and played with robots. In those events, we needed to tune the height of the LRF for children because the robot’s tracking system was developed for adults.

Therefore, robots with human-tracking functions intended for general public use should be robust to various user traits, including height.

### 2.2. Human Tracking Using a 3D LiDAR

A 3D LiDAR observes the environment in multiple planes. Since a 3D LiDAR obtains more information than an LRF, it is expected to enable more robust and accurate human tracking.

Human detection methods using a 3D LiDAR [16,17,18,32] first obtain a point cloud of the environment, segment the point cloud into individual objects, and classify the objects to detect humans. For example, the method by Goméz et al. [18] first detects the moving part of the point cloud and then creates voxels from the cloud. Then, the voxels are segmented and classified as humans or other objects. Although Goméz et al.’s method requires less computation than similar methods, it still involves voxelization and voxel segmentation, which are costly. The problem with these methods is that human detection is computationally expensive, even when relatively inexpensive machine learning techniques are employed.

## 3. Method

### 3.1. Overview

The problem with the LRF method is that an LRF observes only one plane. A two-dimensional scan is enough for human tracking if the observation plane is appropriate for capturing the human body. Here, “appropriate” means that the plane includes the body part the tracking method aims to capture, such as the torso. However, it is impossible to find an appropriate plane without knowing the shape of the human body. As described in the previous section, the body part captured by the plane changes with different heights. If a human is taller than the expected height, the plane captures the arms, which makes tracking unstable. Conversely, if a human is shorter, the plane captures the head, which causes detection failure. Therefore, the proposed method aims to generate an appropriate 2D scan from the 3D point cloud. After obtaining the 2D scan, we apply the existing human-tracking method for 2D LRFs [24]. Converting a 3D point cloud to a 2D map is not a novel idea. Yoon et al. proposed a method to generate a 2D floor map from a 3D point cloud [33]. However, no previous studies have used 2D-converted point clouds for human tracking.

**Figure 2:**
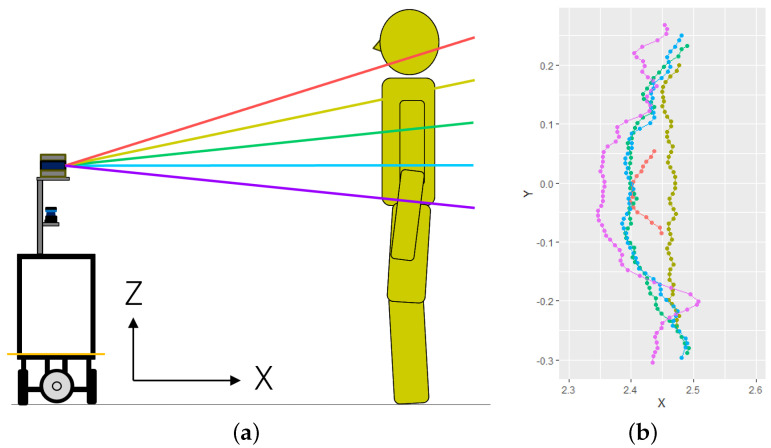
Observation by a 3D LiDAR. The 3D LiDAR scans beams in multiple planes, producing sets of 2D scans. (**a**) Observation by a 3D LiDAR. (**b**) Example of point clouds projected onto a horizontal plane. Different color indicates the measurement by beams with different elevation angles shown in (**a**).

Figure 2 shows the use of a 3D LiDAR. As shown in Figure 2a, a 3D LiDAR casts beams into the environment at several planes (shown in different colors in the figure). As a result of the measurement, it obtains the distance r(θ,ϕ) for a specific azimuth θ∈Θ and elevation ϕ∈Φ, where Θ and Φ are sets of discrete angles. This observed point can be converted to a Cartesian coordinate (x,y,z) as follows:

(1) x=rcosθcosϕ

(2) y=rsinθcosϕ

(3) $$z=h_{s}+r\sin\phi$$

where $h_{s}$ is the height of the sensor.

When we project the observed points onto the X-Y plane, we obtain the points on the plane, as shown in Figure 2b. Here, we have multiple points for a specific azimuth θ. Then, we choose the nearest point in that azimuth.

(4) $$\hat{r}\left(\theta \right)=\min_{\phi \in V}r\left(\theta ,\phi \right)\cos\phi$$

Here, V⊆Φ is the set of elevation angles for projection, which is a subset of all elevation angles.

The problem is how to determine the vertical range of the projection *V*. If we project all points (i.e., V=Φ), human tracking may become unstable because of arm movements. Conversely, when observing only a single horizontal plane, even if it corresponds to the most stable body region such as the chest, changes in height due to leg bending can cause the system to detect the neck or head, resulting in unstable tracking. By slightly expanding the observation volume downward and projecting the resulting point cloud onto a plane, the system becomes more robust to vertical body movements. Therefore, we determine the observation range from the top of a person’s head using a fixed ratio of their height, and the point cloud within that range is projected in two dimensions. When the person’s height is *h* and the ratio is $0<r_{obs}<1$, then

(5) $$V=\{\phi |\phi \in \Phi \;\mathrm{and}\;h\cdot r_{obs}\leq h_{s}+D\sin\phi \leq h\}$$

where *D* is the distance between the 3D LiDAR and the point on the object.

To determine *V*, we need to measure the person’s height. Since this method is designed for a human-following robot, we assume that the person is first registered, after which the robot starts following them. When registering the person, the robot measures the height of that person.

### 3.2. Measurement of Human Height

**Figure 3:**
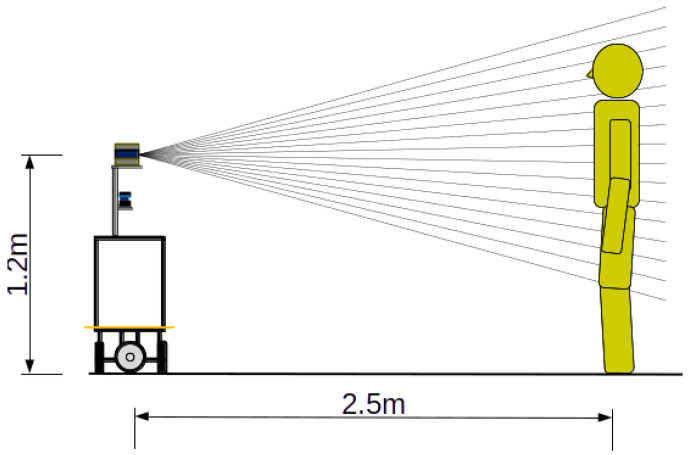
Measurement of human height. The target person first stands 2.5 m in front of the robot. Then, the robot measures the person’s height using a 3D LiDAR.

Figure 3 shows the measurement of a person’s height. It is assumed that the robot tracks a single pre-registered target. The target person stands 2.5 in front of the robot, and the robot measures the person’s height using a 3D LiDAR. Since the approximate distance between the robot and the person is known, the person’s height can be determined by identifying the highest elevation angle that detects an object around 2.5 m away. When registering the person, it is possible that the robot cannot maintain the required distance from the person. We do not address this case in this paper. Instead, we assume that the person is observed at a pre-determined height. Once the robot can measure the height of the person, the proposed method is applied to improve measurement accuracy.

Since the 3D LiDAR is installed at a height of 1.2 m ($h_{s}=1.2$ m) and has an elevation angle range of ±15 degrees, the measurable height range is given by $1.2\pm 2.5\tan\left(15\pi /180\right)=\left[0.53,1.87\right]$ m.

### 3.3. Human Following

After obtaining the point cloud, the points are projected onto a plane using Formula (4). After that, the 2D point cloud data are treated in the same way as measurements by a 2D LRF.

We used the human-following method by Hiroi et al. [19,24]. We briefly describe the method.

**Figure 4:**
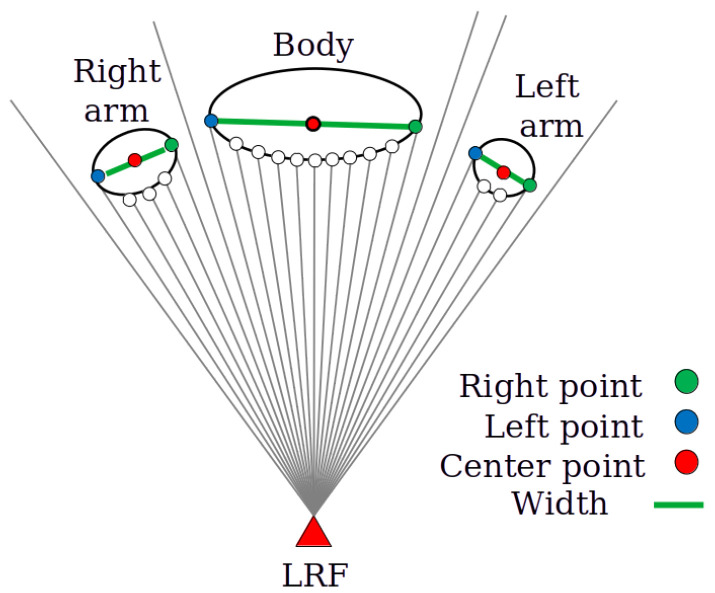
Overview of the human detection method. We segment the observation into objects using an LRF based on the measured distances and identify human bodies based on the widths of the objects.

Figure 4 shows the detection of the human body. When we obtain the observation $\hat{r}\left(\theta_{i}\right),\;i=1,\ldots ,N$, we calculate the distance difference, $\Delta D_{i}=\left|\hat{r}\left(\theta_{i}\right)-\hat{r}\left(\theta_{i+1}\right)\right|$, and detect the range, [j,k], where $\Delta D_{i}<D_{th}$ for j≤i≤k, $\Delta D_{j-1}>D_{th}$, and $\Delta D_{k+1}>D_{th}$. Here, $\theta_{j}$ and $\theta_{k}$ are the rightmost and leftmost angles of the object. $D_{th}$ is the object detection threshold, and we use $D_{th}=0.15$ m in a later experiment. Then, we calculate the width of the object as

(6) $$W=\sqrt{\hat{r}{\left(\theta_{j}\right)}^{2}+\hat{r}{\left(\theta_{k}\right)}^{2}-2\hat{r}\left(\theta_{j}\right)\hat{r}\left(\theta_{k}\right)\cos\left(\theta_{k}-\theta_{j}\right)}.$$

We regard the object as a candidate for a person when 100≤W≤800 mm, and the center point of a candidate is the midpoint of the line segment between the leftmost and rightmost points [24].

After detecting the coordinates of the candidates, the robot determines the target person to follow. Let (x(i,t),y(i,t)) be the coordinates of the *i*-th candidate in the robot’s coordinate system at time *t*, and let p(t) be the index of the target person among the candidates at time *t*. The origin of the robot’s coordinate system is the position of the LRF. Then, we determine p(t) as follows:

(7) Δx(i,t)=x(i,t)−x(p(t−1),t−1)

(8) Δy(i,t)=y(i,t)−y(p(t−1),t−1)

(9) $$\mathrm{inarea}\left(\Delta x,\Delta y\right)=\left\{\begin{array}{cc} \mathrm{True} & \sqrt{\Delta x^{2}+\Delta y^{2}}<R_{min}\\ \mathrm{False} & \mathrm{otherwise} \end{array}\right.$$

(10) $$p\left(t\right)=\arg\min_{i}x{\left(i,t\right)}^{2}+y{\left(i,t\right)}^{2}\;\text{s.t.}\;\mathrm{inarea}\left(\Delta x\left(i,t\right),\Delta y\left(i,t\right)\right)$$

These formulas indicate that we choose the nearest candidate to the LRF who is not too far from the target person’s location at the previous time step. We used $R_{min}=0.6$ m.

After determining the target person, we control the rotation of the robot’s wheels to keep a constant distance from the target [19,24].

## 4. Experiment

### 4.1. Height Measurement

We experimented with measuring human height using a 3D LiDAR. We used a Velodyne VLP-16-LITE (https://www.mapix.com/lidar-scanner-sensors/velodyne/velodyne-vlp16-lite/, accessed on 1 February 2025) as the 3D LiDAR. Table 1 shows the specifications of the VLP-16-LITE.

**Figure 5:**
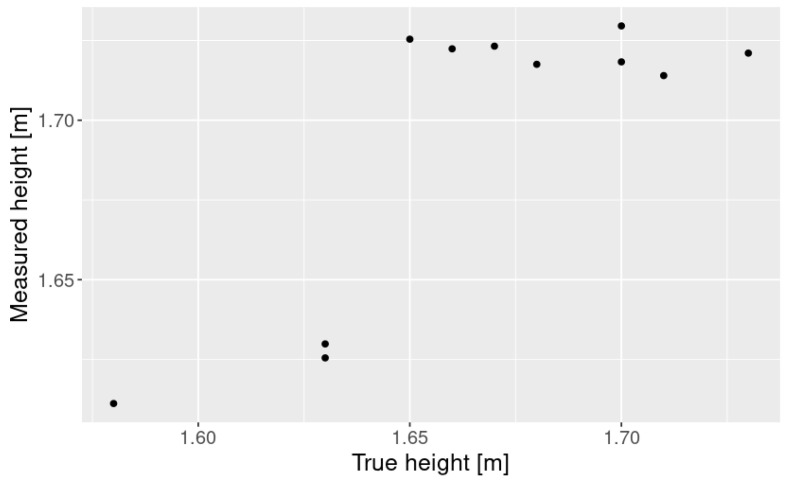
Results of height measurement.

Figure 3 shows the participants standing 2.5 m in front of the 3D LiDAR. We invited eleven participants, whose heights ranged from 1.58 to 1.73 m. Figure 5 shows the measurement results. Since the elevation angles are discrete, the measured height values are also discrete. The average RMS error was 3.83 cm.

### 4.2. Optimal Projection Range

Next, we investigated the optimal projection range. As described in Formula (5), the range is determined by the ratio $r_{obs}$. Therefore, we carried out human-following experiments with different values of $r_{obs}$ and observed the stability of human size. Human size and position estimation are stable if the projection range is appropriate.

**Figure 6:**
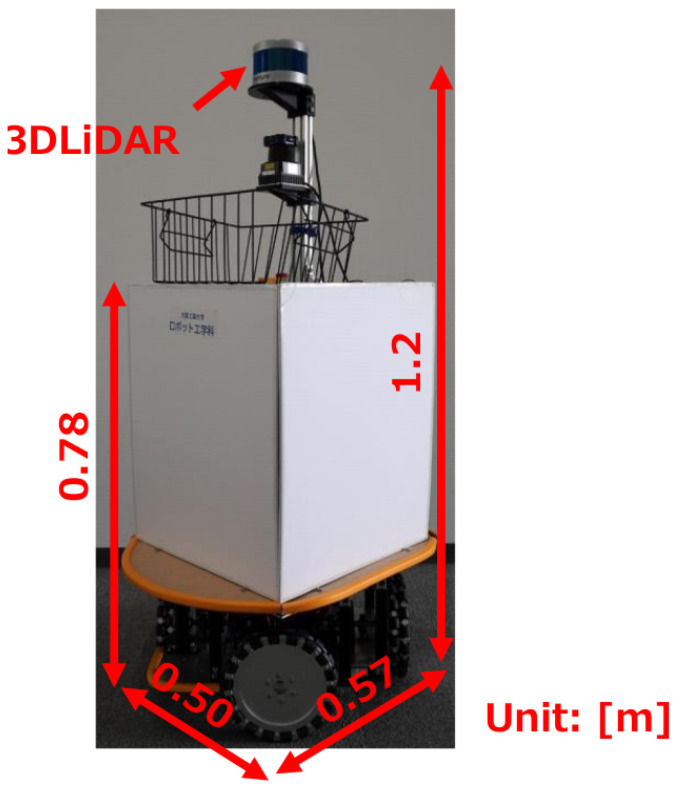
The robot used in the experiment.

Figure 6 shows the robot. The dimensions of the robot are 0.50×0.57×1.2 (WDH m), and its weight is 25 kg. The robot is equipped with four omni wheels to enable omnidirectional movement. The tread is 0.45 m, and the maximum translation speed is 1.4 m/s. The 3D LiDAR is installed at a height of 1.2 m. The 3D LiDAR observes objects at 10 Hz.

The specifications of the PC controlling the robot were as follows: an HP Pavilion Gaming Laptop 15-dk0000 (HP Japan Inc., Tokyo, Japan), with an Intel Core i7-9750H CPU 2.60 GHz CPU (Intel Corp., Santa Clara, CA, USA), and 16 GB of memory. The OS was Ubuntu 20.04.6 LTS with ROS Noetic.

**Figure 7:**
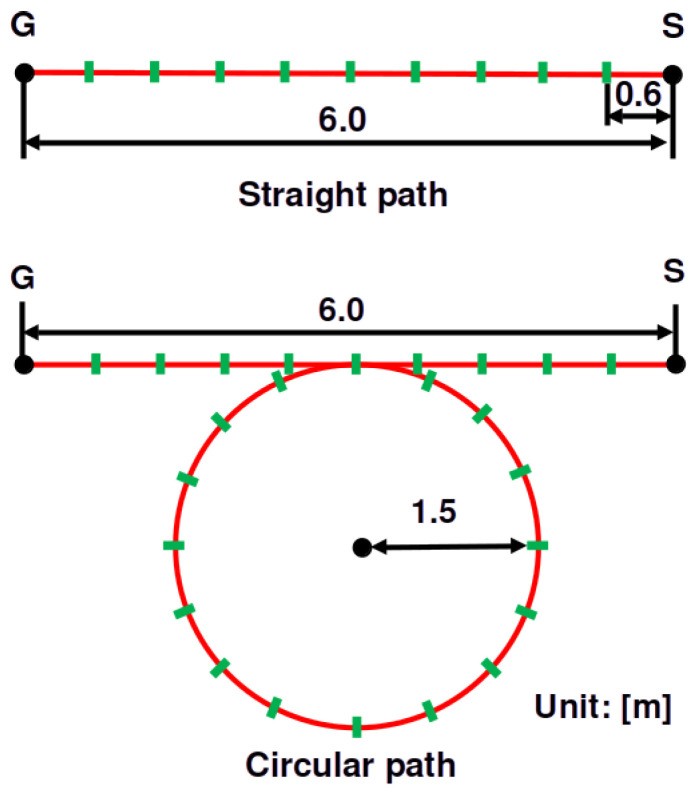
The walking paths used in the experiment. We used a straight path (**upper** figure) and a circular path (**lower** figure).

In this experiment, three participants walked along the paths shown in Figure 7. We chose participants with various heights. The participants’ heights were 1.58, 1.70, and 1.83 m. We prepared two paths: a straight path and a circular path. We marked every 0.6 m on the path, and the participants stepped on the marks synchronously with a metronome sound. The metronome’s speed was 120 BPM; thus, a participant’s walking speed was 1.2 m/s. The robot moved behind the walking participants and measured them. This experiment used four projection ranges: $r_{obs}\in \left\{0.2,0.3,0.4,0.5\right\}$. Thus, we conducted 24 experiments (three participants × two paths × four ranges).

After the experiment, we investigated the results from two points of view: the stability of the human figure and the stability of human tracking. As described in the previous section, an object’s width is the key to detecting a human. Therefore, human tracking becomes difficult if human width estimation is unstable.

**Figure 8:**
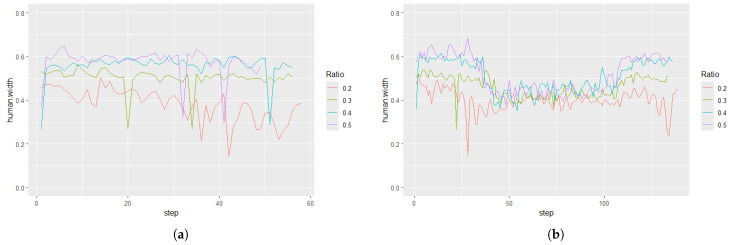
Estimation results of human width (Participant 2). (**a**) Straight path. (**b**) Circular path.

Figure 8 shows the human width estimation results for Participant 2. When $r_{obs}=0.2$, the estimated width was narrower than that with other values of $r_{obs}$. When a participant walked along a circular path, the following robot observed the participant from an angle, making the observed width narrower than when observed from the front.

**Figure 9:**
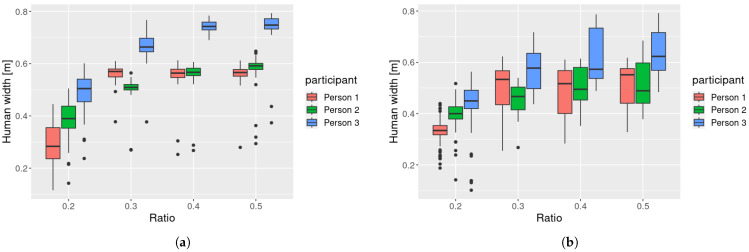
Distribution of human widths. (**a**) Straight path. (**b**) Circular path.

Figure 9 shows the distribution of the estimated human widths. The figure indicates that the estimated human width with $r_{obs}=0.2$ was narrower than under the other conditions for all participants, suggesting that the head width was measured when $r_{obs}=0.2$, while the body width was measured under the condition $r_{obs}\geq 0.3$. We conducted an ANOVA, considering the ratio, participants, and paths as factors. The results showed that all factors were statistically significant at the 1% level. Then, we further conducted a Tukey honest significant difference test to compare pairwise significance and found 5% significant differences between the ratios 0.4 and 0.5 and 1% significant differences between all other conditions.

Table 2 shows the mean and standard deviation of the human width measurements. The standard deviation values are related to the stability of human measurement, which is important for human tracking. The standard deviation decreased when $r_{obs}$ was small. One possible reason for the increased standard deviation at large $r_{obs}$ was the influence of arm movements. According to the average human body size [34], the average ratio of head-to-axilla length to height is 0.264 for males and 0.260 for females. Therefore, if $r_{obs}$ exceeds this value, the arms are included in the observed body, causing the measured body width to fluctuate with arm movements.

We conducted a statistical test based on these results. First, we applied Bartlett’s test to examine whether the variances in these categories were different. The results confirmed statistically significant differences among these ratios ($p=1.93\times 10^{-10}$). As a post hoc test, we conducted an F-test with Bonferroni correction, as shown in Table 3. The results showed that the conditions $r_{obs}\in \left\{0.2,0.3\right\}$ and $r_{obs}\in \left\{0.4,0.5\right\}$ formed distinct groups, and their variances were significantly different.

From these observations, we decided that the optimal value of $r_{obs}$ was 0.3.

### 4.3. Human-Tracking Experiment with Various Paths

**Figure 10:**
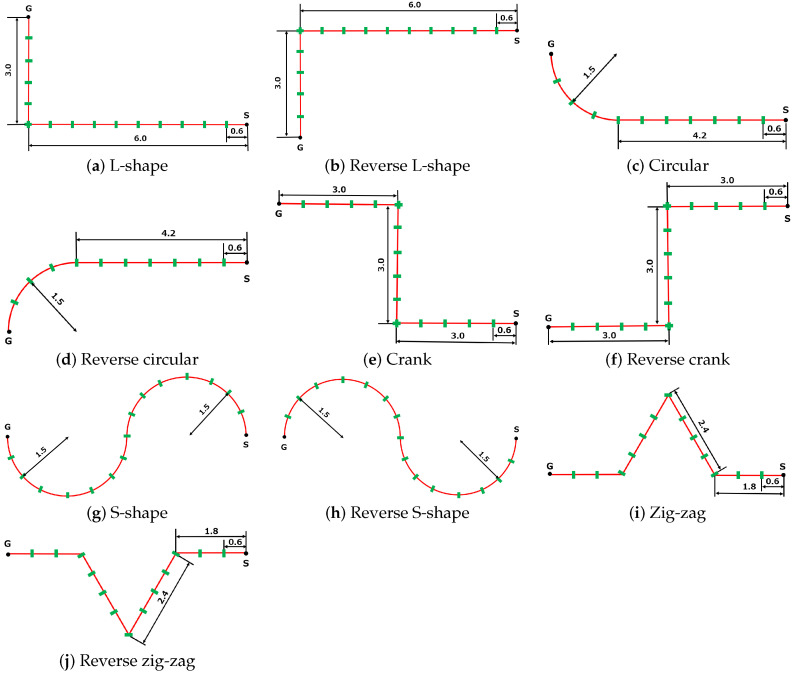
The ten paths examined in the experiment.

Next, we conducted an experiment comparing the optimal $r_{obs}$ value ($r_{obs}=0.3$) with the condition in which the entire point cloud was used (i.e., $r_{obs}=1$) across ten different paths. Figure 10 shows the paths used in the experiment. The experimental paradigm and participants were the same as those in the previous experiment.

**Figure 11:**
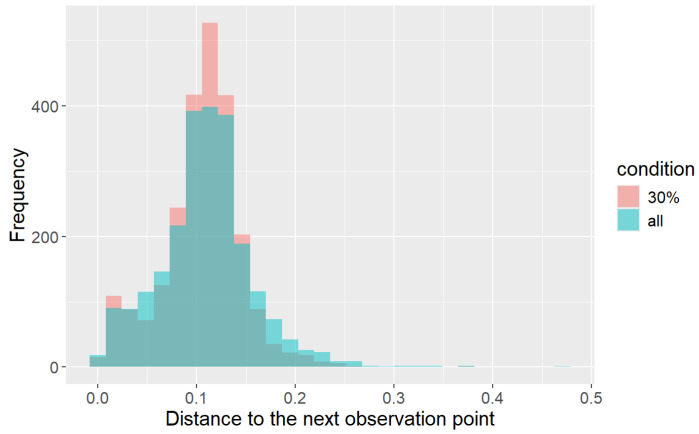
The distribution of distances between contiguous observation points. Two histograms with different colors are superimposed.

**Figure 12:**
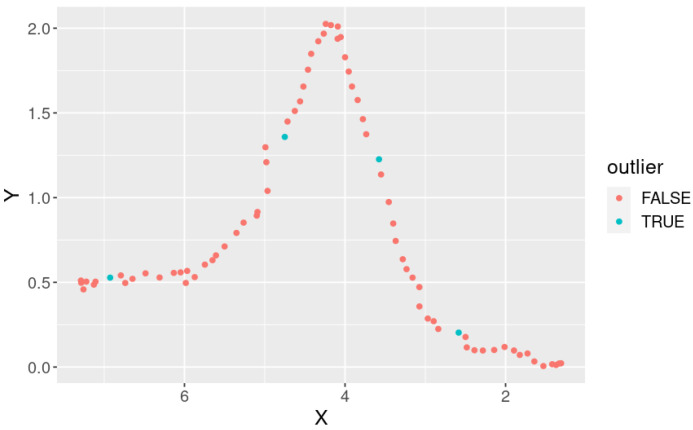
An example of observed points: the path was “zig-zag”, the participant was “Person 3”, and the entire point cloud was used ($r_{obs}=1)$.

We counted outlier points in the measured human positions as an evaluation metric. Here, we measured the distance between contiguous human positions. Since the walking speed of a participant was 1.2 m/s and the observation frequency was 10 Hz, the distance between the contiguous points should be 0.12 m. However, the distance fluctuated because of the measurement error. Figure 11 shows the distribution of the distances. In this figure, two histograms (pink for the “30%” condition ($r_{obs}=0.3$) and blue for the “all” ($r_{obs}=1$) condition) are superimposed. We used all data (three participants and ten paths) to calculate the histogram. We can see that the distribution for the “30%” condition is more concentrated at 0.12 m and has fewer outliers than the “all” condition. Here, we regard points with distances over 0.2 m as outliers. Figure 12 shows an example of the observed points with outlier points. The outlier points happened because of the effect of arm motion. When one or both arms were regarded as part of the body, the center position of the body fluctuated with the arm motion.

**Figure 13:**
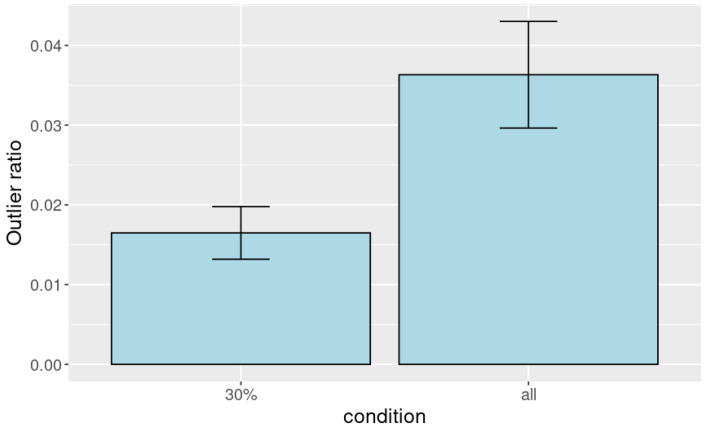
Ratio of outlier points to all the observed points. The error bars show the standard error.

Figure 13 shows the distribution of the outlier point ratio among all observed points. This figure shows that the ratio of outliers using a 30% point cloud from the top of the head (1.65%) was smaller than that using the entire point cloud (3.63%). We tested the difference with a single-sided unpaired *t*-test. As a result, we found a statistically significant difference between the conditions (p=0.0055).

**Figure 14:**
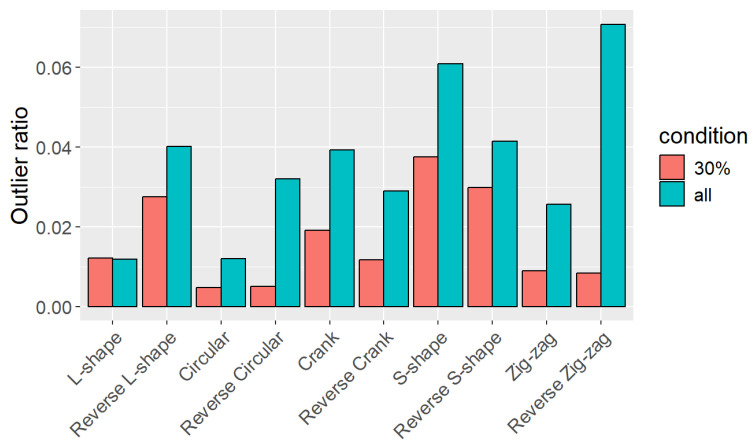
Ratio of outlier points for each path.

Figure 14 shows the outlier ratio of each path. The figure shows that the proposed condition (30%) resulted in fewer outlier points than using the entire point cloud, except for the L-shaped path. The outlier ratio varied across paths, with more complex paths, such as zig-zag or S-shaped ones, tending to produce more outliers.

## 5. Discussion

### 5.1. Robustness of Human Tracking

In the previous sections, we conducted two human-tracking experiments. In the first experiment, we examined two paths and determined the optimal $r_{obs}$ value based on the stability of the observed human width. In the second experiment, we used ten paths to compare two conditions, with $r_{obs}=0.3$ and $r_{obs}=1$. Although we used different measures for comparison, both measures are closely related to the stability of human tracking.

**Figure 15:**
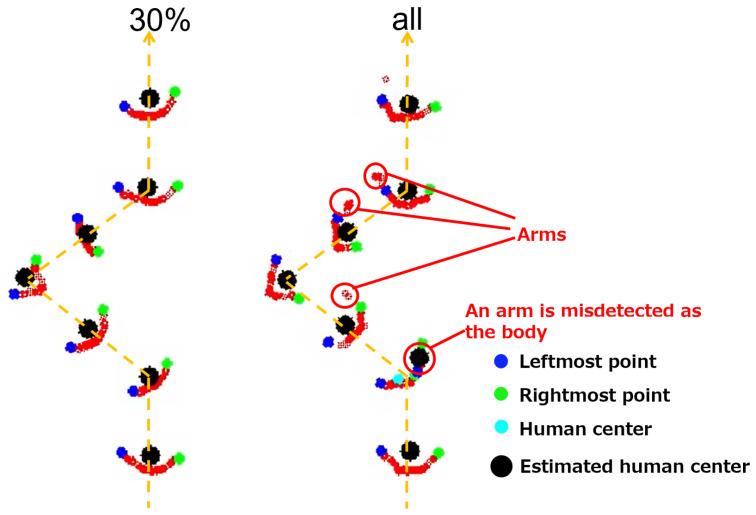
Examples of point clouds of walking people. We can see that the arms are included in the point cloud of the human and misdetected as the body when the entire point cloud is projected. These misdetections can be avoided by using 30% of the point cloud.

Figure 15 shows an example of point clouds of walking people observed by both methods. We can confirm that the arms were detected as part of the point cloud in the “all” method, and those clouds were misdetected as the body. In this situation, the body width suddenly became smaller, and the distance from the previous human position fluctuated. These misdetections did not appear in the “30%” condition.

### 5.2. Computational Efficiency

One advantage of the proposed method is its computational efficiency. Methods that use 3D point clouds directly to detect and track humans often employ neural networks, which makes them computationally expensive. For example, the method by Yin et al. tracks humans at 11 to 16 frame/s using a computer with an Intel Core i7 CPU (Intel Corp., Santa Clara, CA, USA) and a Titan RTX GPU (NVIDIA Corp., Santa Clara, CA, USA) [32], which means their method requires 60 to 90 ms to process one frame. On the other hand, we tested our method on an Intel Core i7-9750H CPU @ 2.60 GHz (Intel Corp., Santa Clara, CA, USA) without a GPU and processed the point cloud in 2.175 ms (1.49 ms for point cloud projection, 0.574 ms for human detection, and 0.111 ms for human tracking).

## 6. Conclusions

This paper introduced a new method for tracking people using a 3D LiDAR. Traditional methods using 2D scanners struggle with people of different heights. Our approach addressed this by projecting the point cloud obtained by a 3D LiDAR onto a 2D plane and then using a standard 2D LRF-based human-tracking method. A key innovation is that we used the top 30% of the 3D point cloud representing the person, making tracking much more reliable regardless of a person’s height.

Through experiments, we found that using the top 30% of the point cloud was the most effective for consistent tracking. Our experiments showed that this method significantly reduced errors and performed well in various scenarios. Specifically, the number of tracking errors was reduced by half compared to using all the 3D data.

The major limitation of the proposed method is the assumption that there is only one target person and that the person is registered before tracking begins. We also assume that registration is performed while the person stands at a certain distance from the robot. We need to develop methods that can relax these assumptions. One idea, as stated above, is for the robot to observe the person at a predetermined plane and then begin using the projected point cloud after observing the entire body of the target person.

We also plan to test this system in more complex environments and with groups of people. Furthermore, we aim to develop a more sophisticated tracking method by considering how people move and their posture. Finally, we will work on making the system faster and more energy-efficient, and we will further explore combining our approach with other sensor data to improve accuracy and robustness. This research offers a robust and efficient way to track people of varying heights, offering more advanced human tracking.

## Figures and Tables

**Table 1:** Specifications of the VLP-16-LITE.

Sensors	16 laser emitters and receivers
Field of View	Horizontal 360 degrees, vertical ±15 degrees
Range	0.1 to 100 m
Sampling frequency	5 to 20 Hz
Sampling speed	≈300,000 point/s
Precision	±3 cm (1σ @ 25 m)
Angle resolution	Horizontal 0.1 to 0.4 degrees, vertical 2.0 degrees

**Table 2:** Mean and standard deviation of human width measurements.

Ratio	Mean [m]	Standard Dev. [m]
0.2	0.392	0.087
0.3	0.532	0.092
0.4	0.564	0.110
0.5	0.579	0.108

**Table 3:** *p*-values from the multiple comparison test of variances between different ratios, with Bonferroni correction.

Ratio	0.2	0.3	0.4
0.3	1.0	-	-
0.4	$1.69\times 10^{-7}$	$2.08\times 10^{-5}$	-
0.5	$2.27\times 10^{-6}$	$1.87\times 10^{-4}$	1.0

## Data Availability

The data are available upon request.
